# Psychological Adjustment and Tolerance for Psychological Pain: A Chain Mediation Model of Stress Mindset and Perceived Stress

**DOI:** 10.3390/bs16010151

**Published:** 2026-01-21

**Authors:** Metin Çelik, Hasan Batmaz, Nuri Türk, Sümeyye Derin

**Affiliations:** 1 Department of Psychology, Siirt University, Siirt 56100, Türkiye; metincelik@siirt.edu.tr; 2 Department of Psychology, Karabük University, Karabük 78050, Türkiye; hasanbatmaz@karabuk.edu.tr; 3 Department of Guidance and Psychological Counselling, Siirt University, Siirt 56100, Türkiye; nuri.turk@siirt.edu.tr; 4 Youth Studies Research & Application Center, Sakarya University, Sakarya 54050, Türkiye; 5 Department of Psychology, Sakarya University, Sakarya 54050, Türkiye

**Keywords:** psychological adjustment, tolerance for psychological pain, stress mindset, perceived stress

## Abstract

Stressful life events can cause individuals to experience psychological pain. Tolerating psychological pain depends on the ability to psychologically adjust to challenging situations and to view stress as enhancing. Therefore, this study aimed to examine the path from psychological adjustment to tolerance for psychological pain in terms of perceived stress and stress mindset. The study sample consisted of 709 adults from Turkey. A hypothetical model was tested using a chain mediation analysis. The study findings indicated that psychological adjustment predicted tolerance for psychological pain. Perceived stress and stress mindset were also found to have a chain mediating effect on the relationship between psychological adjustment and tolerance for psychological pain. The results are expected to contribute to programs and practices developed by mental health professionals to improve tolerance for psychological pain. These practices may specifically aim to increase psychological adjustment and an enhancing stress mindset.

## 1. Introduction

Increased uncertainty, wars, disasters, and global pandemics confront individuals with challenging stressors in today’s world. The World Economic Forum’s 2025 Global Risks Report highlights thirty-three different risk factors that will affect societies in the next two to ten years, including armed conflicts, extreme weather events, and social polarization (World Economic Forum, 2025). Conflicts around the world have reached their highest level in the last 25 years. A total of 421 million people have been affected by these conflicts, and this number is projected to reach 435 million by 2030 (World Bank, 2025). In 2025, more than 300 million people worldwide were affected by health crises (World Health Organization, 2025).

Turkey has also faced large-scale stressors in recent years, mirroring global developments. These include economic instability, proximity to active conflict zones, and the earthquake described as the catastrophe of the century. In the 6 February 2023 earthquake centered in Kahramanmaraş, over 50,000 people lost their lives and over 100,000 were injured (Republic of Türkiye Ministry of Interior, 2024). Over 700,000 people have experienced internal displacement in eleven provinces affected by the earthquake (Turkish Statistical Institute, 2024). These challenging life events and high-stress environments can shape individuals’ stress assessments and affect their psychological functioning. Indeed, the results of a meta-analysis study examining the psychological effects of the February 6th earthquake showed that post-traumatic stress disorder (PTSD), depression, and anxiety symptoms were common in individuals who experienced the earthquake, and the severity of PTSD symptoms remained high for a year (Çınaroğlu et al., 2025). Furthermore, economic uncertainties (Sarı et al., 2024) and proximity to areas experiencing armed conflict (Hoffmann et al., 2024) have been associated with the prevalence of mental health disorders in the community.

Stressful life events, physical factors, illness, and negative cognitions can all result in psychological suffering (S. Chen et al., 2023). Psychological pain is defined as the emotional state experienced by an individual when they realize the discrepancy between their actual self and their ideal self (Joffe & Sandler, 1967). Psychological pain is known to have significant relationships with feelings of failure, abandonment, emotional distress, and emptiness (Orbach et al., 2003). Similarly, when the severity of psychological pain increases and becomes chronic, it can become pathological (Ducasse et al., 2017; Sehlikoğlu et al., 2024). Furthermore, as the level of psychological pain increases, the risk of suicide increases (S. Chen et al., 2023; Levi-Belz et al., 2019). When psychological pain and strain become unbearable, suicide may be seen as the only way to end the pain (Shelef et al., 2015; Türk et al., 2025a). In particular, when an individual cannot cope with the feeling of being trapped, vulnerable, and having emotional problems caused by the pain they experience, they may choose to kill themselves to escape (Arslan et al., 2024; Türk et al., 2024a; Türk et al., 2024b). At this point, the concept of psychological pain tolerance, which refers to the ability to manage pain (Meerwijk et al., 2019), becomes important.

Tolerance for psychological pain is the adaptive response to psychological pain that occurs during stressful times (Meerwijk et al., 2019). Positive and significant relationships have been found between tolerance for psychological pain and emotional regulation skills, self-esteem, optimism, meaning in life, mindfulness, and life satisfaction (Becker et al., 2019). In addition, tolerance to psychological pain plays a critical role in preventing pathological events such as suicide (Osaki et al., 2021) that may result from stressful life events (Sehlikoğlu et al., 2024). Furthermore, it is necessary to strengthen tolerance for psychological pain when exposed to the devastating effects of epidemics, economic crises, and earthquakes (Türk et al., 2025b). The key issue here is how to establish a life adaptation process that will build resilience after a stressful life event (Batmaz et al., 2021; Doğrusever et al., 2022). Considering the current global and national context, examining the processes of assessing and perceiving stress in the face of challenging life events in relation to protective psychological mechanisms is particularly significant. Therefore, this study examined the mediating roles of stress mindset and perceived stress in the relationship between psychological adjustment and tolerance to psychological pain.

### 1.1. Psychological Adjustment and Tolerance for Psychological Pain

Adjustment involves the psychological processes that enable people to cope with the demands and challenges of daily life (Weiten et al., 2011). Psychological adjustment is also defined as an individual’s capacity to manage stressful situations, anxiety, and depressive symptoms (Cruz et al., 2020). According to Black and Gregersen (1991), adjustment is the level of psychological comfort an individual exhibits when faced with a new situation. Individuals with high levels of adjustment perceive reality appropriately, are able to control their behavior, and develop close relationships with others (Smith et al., 2016). These individuals also have higher levels of psychological well-being. Because they do not have difficulty adapting to stressful situations, they are less likely to experience mental disorders and negative emotions (Bantjes & Kagee, 2018; Satıcı et al., 2024; Sun et al., 2023). According to Rohner (1975), general psychological adjustment is achieved through seven personality traits that emerge as a result of the interaction between parental acceptance and rejection perceived during childhood. One of these dimensions is emotional stability. Emotional stability is the ability to be psychologically balanced and not become overwhelmed when faced with difficulties and stressful situations (Rohner et al., 1978). Individuals with high emotional stability are not afraid to encounter new situations. They can demonstrate effective coping behaviors in emergencies (Özgüven, 1992). Emotional stability, particularly the ability to achieve balance in stressful situations, is a crucial component of psychological adjustment (Rohner et al., 1978).

Considering theoretical knowledge about psychological adjustment, it can be said that individuals with high levels of psychological adjustment may have a high pain tolerance. As a matter of fact, a study found a significant relationship between psychological adjustment and pain tolerance (Tehrani et al., 2020). In particular, both psychological adjustment and tolerance for psychological pain share common characteristics, such as facilitating stress coping. It is also known that individuals with dysfunctional coping skills in challenging experiences such as depression may be less able to tolerate psychological pain (Yeşiloğlu et al., 2023). Therefore, individuals who cannot psychologically adjust to challenging experiences may have a lower tolerance for pain and its management. Furthermore, some studies have found significant relationships between psychological resources, which are closely related to adjustment and used to measure adjustment (Birman et al., 2014; Rohner et al., 1978; Zheng et al., 2004), and tolerance for psychological pain. For example, one study found significant positive correlations between tolerance for psychological pain and emotional regulation skills, self-esteem, optimism, meaning in life, mindfulness, life satisfaction, and positive emotionality (Becker et al., 2019). Therefore, based on the literature cited above, psychological adjustment is thought to be one of the predictors of tolerance for psychological pain.

### 1.2. Stress Mindset and Perceived Stress

Research findings have proven that stressful life events have negative effects on an individual’s mental health (Bjørndal et al., 2024; Lindert et al., 2020). However, in recent years, it has been argued that the impact of stressful life events may be related not only to external environmental factors but also to an individual’s cognitive evaluations of stress, and the concept of stress mindset has been proposed (Crum et al., 2013). Stress mindset refers to metacognitive processes that involve positive or negative evaluations of the consequences of stress (Journault & Lupien, 2024; Keech & Hamilton, 2019). The focus in these interpretations is not on the magnitude of the stress but on whether it is debilitating or enhancing (Crum et al., 2017). Those who believe that the overall consequences of stress will be negative have a debilitating stress mindset. Conversely, those who believe that stress can have positive effects have an enhancing stress mindset (Huebschmann & Sheets, 2020). Individuals with an enhancing stress mindset believe that stress has a positive impact on learning, growth, health, and resilience (Crum et al., 2013). They do not see stressful life events as a threat and think that these events, even though they are challenging, improve them (Mansell, 2021). Individuals with a debilitating stress mindset believe that stress will harm their performance, productivity, health, and resilience (Crum et al., 2013). Accordingly, the stress mindset essentially puts forward the view that stress can be not only debilitating but also developmental, in relation to individuals’ evaluations (Silva et al., 2023).

The stress mindset plays a crucial role in shaping cognitive, emotional, and physiological responses to challenging and threatening stress situations (Crum et al., 2017). As a matter of fact, those with a debilitating stress mindset are at greater risk of burnout (Klussman et al., 2021) and negative affect (Laferton et al., 2020). On the other hand, a constructive stress mindset is known to alleviate the development of symptoms of depression and anxiety (Huebschmann & Sheets, 2020), is associated with fewer PTSD symptoms following traumatic life events (Williams & Ginty, 2024), and predicts post-traumatic growth (Zhang et al., 2023). A recent study determined that a developmental stress mindset was a predictor of longer sleep duration and better sleep quality after one year compared to a debilitating stress mindset, acting as a buffer against the negative impact of stress levels on sleep (Zhao et al., 2025). Another study found that the interaction between personality traits and stress mindset affected individuals’ emotions. Accordingly, neuroticism and a debilitating stress mindset weakened the link between life stress and positive emotions. On the other hand, the interaction between agreeableness, extraversion, openness, and conscientiousness personality traits and a developmental stress mindset weakened the link between life stress and negative emotions (Zeng et al., 2024). A study conducted by Park et al. (2019) with adolescents showed that the negative relationship between negative life events and perceived distress was weaker in those who adopted an enhancing stress mindset. Adolescents with a debilitating stress mindset reported higher levels of distress in the face of adversity. Another study found that stress mindset predicts distress tolerance (Alker, 2019). Thus, the literature suggests a significant relationship between stress mindset and tolerance for psychological pain. Indeed, individuals with an empowering stress mindset describe stressful life events as enhancing (Mansell, 2021). This may positively impact their ability to endure and withstand the psychological pain that may arise from challenging stressful events. However, to the authors’ knowledge, no studies in the literature have focused on the relationship between stress mindset and tolerance for psychological pain. Therefore, it is believed that this study will greatly contribute to the literature on the relationship between stress mindset and tolerance for psychological pain.

Another concept thought to be related to the stress mindset is psychological adjustment. Both stress mindset and psychological adjustment constitute components of the stress process, but they are concepts that point to different temporal contexts of stressful situations. In this context, stress mindset refers to the belief system that an individual adopts regarding the outcome of stress (Keech & Hamilton, 2019), while psychological adjustment refers to the individual’s capacity to manage stressful situations (Cruz et al., 2020). Therefore, it can be stated that stress mindset is related to the evaluation processes before and during challenging life events; and psychological adjustment is related to the adjustment processes that occur during and after these events. One of the seven components of psychological adjustment, worldview consists of an individual’s positive or negative assessments of life. Individuals with a negative worldview evaluate life and situations as dangerous or untrustworthy (Rohner & Britner, 2002). However, individuals with a positive worldview evaluate life as fundamentally good, safe, happy, and non-threatening (Rohner, 1986). Similarly, individuals’ positive or negative appraisal of stress determines their confidence in life and perceived happiness. According to Rohner (1975), another dimension of general adjustment is self-efficacy. One study found that self-efficacy predicts the stress mindset. In other words, individuals with high self-efficacy evaluate stressors as beneficial (Subhasree et al., 2023). Indeed, according to Başaran (2005), people with high levels of adjustment are more likely to approach stressful situations more positively. There is a negative correlation between psychological adjustment and perceived stress (Çetiner et al., 2018; Turgut et al., 2020), and individuals with high levels of psychological adjustment can manage stressful situations (Cruz et al., 2020). This prevents them from seeing stress as something uncontrollable and overwhelming. Therefore, considering theoretical knowledge and research, it can be argued that stress mindset may play a mediating role between psychological adjustment and tolerance for psychological pain.

Another variable that may mediate the relationship between psychological adjustment and tolerance to psychological pain is perceived stress. The perceived stress is the degree to which an individual evaluates the level of stress they experience as overwhelming and uncontrollable (Cohen et al., 1983). As perceived stress involves an individual’s subjective appraisal of stressful situations (Lazarus & Folkman, 1984), it determines the degree to which they are affected by stressful situations. Unlike stress mindset and psychological adjustment, perceived stress refers to individuals’ appraisal of challenging situations as they occur and indicates the extent to which these experiences are perceived as controllable and manageable (Cohen et al., 1983). Studies have found positive significant relationships between stress and psychological symptoms such as anxiety, depression, somatization and hostility (Onan et al., 2015), screen addiction (O. Yıldırım et al., 2026), burnout (Ruiz-Fernández et al., 2020), and suicide risk (Ballabrera et al., 2024; Elbogen et al., 2021). Furthermore, negative and significant relationships were found between perceived stress and hope and life satisfaction (Hatun & Kurtça, 2024), psychological pain tolerance (Becker et al., 2019), and stress mindset (Türk & Çelik, 2024). Furthermore, significant negative relationships have been identified between perceived stress and five dimensions of distress tolerance (uncertainty, ambiguity, disappointment, negative emotion, and physical discomfort) (Bardeen et al., 2017). Similarly, perceived stress, which represents a negative evaluation of stress, is known to reduce tolerance for psychological pain (Becker et al., 2019).

Perceived stress, which predicts tolerance for psychological pain, is thought to be predicted by psychological adjustment. Indeed, psychological adjustment is defined as an individual’s ability to effectively cope with the demands of environmental conditions and the stress created by these demands (Seaton, 2009). Perceived stress, on the other hand, evaluates stressful life events as unmanageable situations that must be avoided. Furthermore, neurotic tendencies are one of the important sub-dimensions of the general level of maladjustment (Özgüven, 1992). Individuals with high levels of neuroticism have higher perceived stress levels. Because these individuals appraise events as threatening, their coping resources, perceived efficacy, and self-regulation skills are insufficient (Lecic-Tosevski et al., 2011). Therefore, it is expected that an increase in an individual’s level of psychological adjustment will play a significant role in reducing perceived stress. Studies supporting this expectation have found significant negative relationships between adjustment and perceived stress (Bretherton & McLean, 2015; Çetiner et al., 2018; Turgut et al., 2020). Therefore, theoretical information in the literature and research indicate that perceived stress may mediate the path from psychological adjustment to tolerance for psychological pain.

### 1.3. The Present Study

According to the Transactional Stress Model, individuals constantly evaluate stimuli from the environment. The intensity of the response to stress is influenced by the cognitive evaluations made by the individual (Lazarus & Folkman, 1984). Cognitive evaluations are associated with the individual’s psychological functioning and tolerance to psychological pain in response to stressful events (Qiang et al., 2025; Spătaru et al., 2024). In this context, the present research is based on the Transactional Stress Model, which considers stress not as an external factor but as a subjective experience.

Individuals with high psychological adjustment are less affected by adverse life conditions, do not give up in the face of obstacles, can effectively cope with stressful situations, and can tolerate distress (Başaran, 2005; Satıcı et al., 2024). Furthermore, because individuals with high levels of adjustment have a positive worldview, they have a positive perspective on life, events, and themselves. Individuals with an enhancing stress mindset also exhibit a positive approach to the stressful life events they experience. In this context, individuals with high psychological adjustment are expected to have an enhancing stress mindset. Furthermore, individuals with a positive stress mindset have been found to have lower perceived stress levels (Park et al., 2019; Sutin et al., 2023; Türk & Çelik, 2024). The negative impact of perceived stress on mental health problems can be exacerbated by a debilitating stress mindset and mitigated by an enhancing stress mindset (Huebschmann & Sheets, 2020). Furthermore, holding negative cognitions in the event of stressful situations can lead to psychological pain (S. Chen et al., 2023). Indeed, according to Becker et al. (2019), as perceived stress increases, tolerance for psychological pain decreases. Consequently, considering the findings in the literature, it is thought that stress mindset and perceived stress may play a chain mediating role in the relationship between psychological adjustment and tolerance for psychological pain.

**Figure 1:**
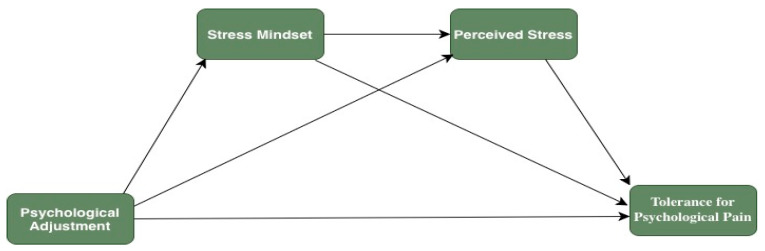
Proposed mediation model.

This study is expected to contribute to a more in-depth understanding of the determinants of tolerance for psychological pain. Identifying the variables associated with tolerance to psychological pain will enable both preventive and therapeutic studies on psychological pain. Therefore, this study was designed to explore the psychological mechanisms underlying the pathway from psychological adjustment to tolerance for psychological pain. The hypothesized chain mediation model is presented in Figure 1. In this context, a hypothetical model was tested using the following hypotheses.

**H1:**  *Psychological adjustment predicts tolerance for psychological pain.*

**H2:**  *Stress mindset will mediate the relationship between psychological adjustment and tolerance for psychological pain.*

**H3:**  *Perceived stress will mediate the relationship between psychological adjustment and tolerance for psychological pain.*

**H4:**  *Stress mindset and perceived stress will have a chain mediation effect in the relationship between psychological adjustment and tolerance for psychological pain.*

## 2. Materials and Methods

In this study, the mediating roles of stress mindset and perceived stress in the relationship between psychological adjustment and endurance of psychological pain were examined using a quantitative correlational method. Participants were selected from easily accessible sampling units due to time, cost, and labor constraints (Büyüköztürk et al., 2014).

### 2.1. Participants

The study data were collected from a total of 709 individuals. The demographic characteristics of the participants, including gender, age, education level, and socioeconomic status, are presented in Table 1.

Participants consisted of 241 males (34%) and 468 females (66%), aged between 18 and 51 years (M = 26.08, SD = 6.41). Regarding educational background, 133 participants (18.8%) had a high school degree, 507 (71.5%) held a bachelor’s degree, and 69 (9.7%) had a postgraduate degree. Based on self-reports, socioeconomic status was described as poor by 126 participants (17.8%), moderate by 547 (77.2%), and good by 36 participants (5.1%).

Participants were recruited using a convenience sampling method through online announcements and social media platforms. Participation was voluntary, and individuals between the ages of 18 and 51 were eligible to take part in the study. The resulting sample consisted of 241 men and 468 women. The higher proportion of female participants reflects a common pattern in online psychological research, where women tend to participate more frequently in voluntary survey studies, particularly in research related to psychological well-being.

### 2.2. Power Analysis

In the power analysis using G*Power (version 3.1.9.7), with an alpha value of 0.05, a medium effect size of 0.15, and a power level of 0.95, the sample size was calculated as 647. After missing data and outliers were removed, the analyses were performed with 709 participants. A subsequent post hoc power analysis revealed that the study’s statistical power was 1.000, confirming that the sample size was more than sufficient for the analysis.

### 2.3. Measures

- Tolerance for Mental Pain Scale (TMPS). This scale is a self-report instrument consisting of 10 items (e.g., “Believe that my pain will go away.”) designed to measure individuals’ tolerance levels for psychological pain (Meerwijk et al., 2019). On this five-point Likert scale, higher scores indicate increased tolerance for psychological pain. The Cronbach alpha value of the scale in Turkish culture is 0.98 (Demirkol et al., 2019). The CFA results for this study are as follows: CMIN/df 4.09, CFI = 0.96, NFI = 0.94, TLI = 0.94 and RMSEA = 0.066. In the present study, the Cronbach α coefficient of the scale is 0.73.
- Brief Adjustment Scale-6 (BASE). The scale was developed to assess psychological adjustment (Cruz et al., 2020) and consists of six items (e.g., “To what extent did you feel unhappy, insecure, and depressed this week?”). The scale is a seven-point Likert (1 = not at all, 7 = extremely) type. As the scores obtained from the scale decrease, psychological adjustment increases (Cruz et al., 2020). Cronbach’s α of the scale in Turkish culture is 0.88 (M. Yıldırım & Solmaz, 2020). The CFA results for this study are as follows: CMIN/df 4.47, CFI = 0.99, NFI = 0.99, TLI = 0.98 and RMSEA = 0.070. Additionally, Cronbach’s α for the scale was found to be 0.90.
- Although the Brief Adjustment Scale–6 was originally developed for university student populations, its core constructs reflect general psychological adjustment processes that are not restricted to student status. In the present study, internal consistency and CFA indicators obtained from the current sample supported the appropriateness of its use with a broader age and occupational range.
- The Perceived Stress Scale. The scale is a 14-item (e.g., “In the last month, how often have you felt nervous and stressed?”) self-report instrument using a five-point Likert format (0 = never, 4 = very often) to assess perceived stress (Cohen et al., 1983). An increase in scores indicates that the individual’s stress perception is high. The scale’s reliability coefficients in Turkish culture are above 0.80 (Eskin et al., 2013). The CFA results for this study are as follows: CMIN/df 4.20, CFI = 0.95, NFI = 0.94, TLI = 0.93 and RMSEA = 0.067. In the present study, Cronbach’s α for the scale was calculated to be 0.82.
- Stress Mindset Measure. The scale is a five-point Likert-type self-report instrument (0 = strongly disagree, 4 = strongly agree) developed to assess individuals’ beliefs about the consequences of stress (Crum et al., 2013). The scale consists of eight items (e.g., “Experiencing this stress depletes my health and vitality.”). An increase in scores indicates an increase in the individual’s level of functional evaluation regarding the consequences of stress. Cronbach’s α and McDonald’s Omega (SPSS 25) reliability values of the scale in Turkish culture are above 0.80 (Türk & Çelik, 2024). The CFA results for this study are as follows: CMIN/df 4.41, CFI = 0.96, NFI = 0.96, TLI = 0.95 and RMSEA = 0.069. In the present study, Cronbach’s α of the scale was calculated to be 0.82.

### 2.4. Procedure

This research was conducted in accordance with the ethical principles of the Helsinki Declaration. Furthermore, the necessary ethical approval was obtained from the Karabük University Social and Human Sciences Research Ethics Committee prior to the research (reference number: E.391045; meeting number: 2024/10; decision number: 72; date of approval: 27 December 2024). The study included individuals aged 18 and over who volunteered. Data were collected through a secure web-based system via an online survey. Participants were recruited through online announcements and social media, and were given informed consent before participating in the study. Incomplete and duplicate responses were excluded from the analysis. Data were collected between 27 March 2024 and 10 May 2024. Only those who agreed to participate were included; others were excluded.

### 2.5. Data Analysis

The study tested a mediation model to examine the mediating roles of stress mindset and perceived stress between psychological adjustment and tolerance for psychological pain. Data from 709 participants, collected voluntarily online via Google Forms, were analyzed using SPSS PROCESS 25 (Model 6). Before the mediation analysis, linearity, normality, and multicollinearity assumptions were assessed. No multicollinearity issues were detected, and the data were normally distributed. Correlation analyses showed acceptable relationships among variables, supporting further analysis. Descriptive statistics, including means and standard deviations, were calculated for all study variables. Indirect effects were tested using a bootstrapping procedure with 5000 resamples and 95% confidence intervals. Statistical significance was evaluated at the *p* < 0.05 level. Results are presented in Table 2.

## 3. Results

Tolerance for psychological pain is positively related to psychological adjustment (*r* = 0.46) and stress mindset (*r* = 0.14), but negatively related to perceived stress (*r* = −0.51) (Table 2).

### Chain Mediational Analyses

Preliminary analyses have shown that psychological adjustment significantly predicts tolerance for psychological pain (Table 3) (confirmed: H1: psychological adjustment predicts tolerance for psychological pain) (*β* = 0.22, 95% CI: 0.10–0.23; *p* < 0.001).

**Figure 2:**
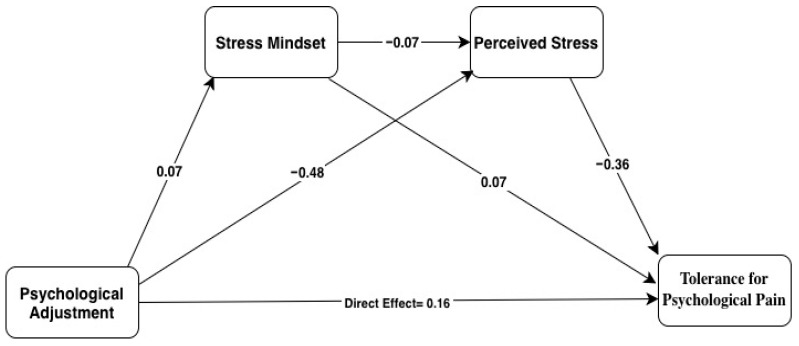
The chain mediation role of stress, mindset, and perceived stress.

The mediation analysis revealed that tolerance for psychological pain was significantly predicted by psychological adjustment (Figure 2 and Table 3) (*β* = 0.16, 95%, LLCI = 0.10 ULCI = 0.23; *p* < 0.01), stress mindset (*β* = 0.07, 95% LLCI = 0.007 ULCI = 0.14; *p* < 0.05) (confirmed H2: stress mindset will mediate the relationship between psychological adjustment and tolerance for psychological pain) and perceived stress (*β* = −0.36, 95% LLCI = −0.45 ULCI = −0.27) (confirmed H3: perceived stress will mediate the relationship between psychological adjustment and tolerance for psychological pain). Finally, mediation analysis revealed that stress mindset and perceived stress played a chain mediating role in the relationship between psychological adjustment and tolerance for psychological pain (confirmed H4: stress mindset and perceived stress will have a chain mediation effect in the relationship between psychological adjustment and tolerance for psychological pain).

The three indirect pathways examined in the study (Ind1, Ind2, and Ind3) showed statistically significant effects on tolerance to psychological pain (Table 3). Collectively, the findings empirically support the proposed chain mediation model. Furthermore, bootstrap estimations based on 5000 resamples also confirm the robustness of the results (Table 4).

## 4. Discussion

The present study aimed to examine the predictive role of psychological adjustment in tolerance for psychological pain, as well as the potential mediating roles of stress mindset and perceived stress in this relationship. Consistent with our hypotheses, the findings revealed that psychological adjustment significantly predicted tolerance for psychological pain, supporting H1. Moreover, stress mindset and perceived stress each mediated this relationship (H2 and H3), and further analysis indicated a significant chain mediation effect (H4), whereby psychological adjustment influenced tolerance for psychological pain through its impact on both stress mindset and perceived stress, respectively. These results highlight the importance of stress-related cognitive and emotional processes in understanding how individuals adapt to and tolerate psychological distress.

According to hypothesis H1, it was predicted that the effect of psychological adjustment on tolerance for psychological pain was significant. Individuals with high levels of psychological adjustment use more flexible, balanced, and problem-oriented coping methods when faced with stressful events in their life. This allows them to make emotional pain more meaningful and to endure it. Psychological adjustment directly relates to the individual’s ability to regulate emotions and thoughts. Thanks to this regulation skill, the individual can tolerate intense psychological pain and prevent it from turning into a crisis. A study conducted by Garnefski et al. (2009) shows that psychological adjustment is closely related to external factors, how the individual evaluates events, and which mental strategies they use. Another study revealed that high psychological adjustment in university students is strongly associated with a decrease in anxiety, stress, depression, and suicidal thoughts, and that social support plays a critical role in increasing these positive effects (Tamizi et al., 2024). The individual’s emotional reactions to the adverse events they experience determine the level of psychological adjustment, and high psychological adjustment serves as an essential factor in coping with negativity. Tolerance for psychological pain may decrease as a result of having or witnessing suicidal thoughts, depression, anxiety and high-stress situations. At this point, strengthening psychological adjustment (such as noticing, accepting and expressing emotions healthily) may lead to increased tolerance and well-being.

In hypothesis H2, the mediating effect of stress mindset on the relationship between psychological adjustment and tolerance for psychological pain was determined. A partial mediating effect of stress mindset, a concept that reduces the direct effect of psychological adjustment on tolerance for psychological pain, was detected. When the stress mindset was included in the model, the impact of psychological adjustment on tolerance for psychological pain decreased. L. Chen et al.’s (2022) study revealed the mediating roles of stress mindset and coping flexibility in the effect of Big Five personality traits on psychological distress, showing that psychological adjustment is affected not only by personality traits but also by the individual’s attitude towards stress and the flexibility of coping strategies. Conejero et al. (2018) emphasize that stress is an essential factor that increases psychological pain, and that this pain increases depression and suicide risk. Therefore, stress management and developing tolerance for psychological pain are of critical importance in terms of psychological adjustment and suicide prevention. The capacity to endure such psychological pain situations as intense discomfort, sadness, distress, loss or trauma that are experienced emotionally and mentally, and to accept and manage this pain, is indirectly affected by the positive stress mindset, which regards stress as an opportunity for development and learning and as a source of energy. Therefore, the effect of psychological adjustment on tolerance for psychological pain is realized through the individual’s ability to transform stress into a positive situation and to think in that way.

The finding that perceived stress mediates the relationship between psychological adjustment and tolerance for psychological pain confirmed hypothesis H3. The positive effect of psychological adjustment on tolerance for psychological pain is indirectly reduced by perceived stress. Perceived stress is related to whether or not the event experienced by the individual is perceived as stressful. In this study, it is seen that perceived stress weakens the effect of psychological adjustment on tolerance for psychological pain. While there is a positive effect of stress mindset in hypothesis H2, there is an adverse effect of perceived stress in hypothesis H3. The existence of a negative relationship between psychological adjustment and perceived stress supports a part of this hypothesis (Bergin & Pakenham, 2016). A study supporting the other part of the hypothesis confirms the positive effect of perceived stress on tolerance for psychological pain, which is proportional to the reaction to depression, anxiety and stress (Miron et al., 2019; Zandifar et al., 2020). Y. L. Chen and Kuo (2020) reveal that high perceived stress in early adolescence increases suicidal behaviors, but psychological resilience significantly weakens this effect and plays a protective role. Based on this, in situations where tolerance for psychological pain is low, such as suicide, the individual’s high adaptive capacity, such as psychological adjustment, has a protective effect.

In the last hypothesis of this study, stress mindset and perceived stress have a chain mediation effect on the relationship between psychological adjustment and tolerance for psychological pain. The impact of psychological adjustment on tolerance for psychological pain decreases first through stress mindset and then through perceived stress (chain mediation). The positive effects of psychological adjustment on stress mindset cause a decrease in perceived stress, which increases tolerance for psychological pain. In this way, the impact of psychological adjustment on tolerance for psychological pain is provided by mediating variables. The effect of psychological adjustment on tolerance for psychological pain emerges indirectly through the individual’s way of thinking about stress and perception of stress. A psychologically adjusted person interprets stressful events more healthily and has a more positive perspective about stress, which reduces their perception of stress and enables them to become more resilient to psychological pain. An individual with low psychological adjustment has beliefs such as “Stress will destroy me” in their stress mindset. This may cause them to perceive events more threateningly and be unable to withstand even the slightest emotional pain. This hypothesis suggests that as individuals’ psychological adjustment increases, they will develop more functional thoughts about stress, resulting in lower stress perception and ultimately greater tolerance for psychological pain.

Psychological adjustment, the ability to cope with stressful situations, anxiety, and depressive symptoms (Cruz et al., 2020), enables individuals to manage the demands of daily life and stressful situations (Weiten et al., 2011). Indeed, according to Satıcı et al. (2024), individuals with high levels of psychological adjustment cope more effectively with stressful situations and are less affected by negative life circumstances (Satıcı et al., 2024). Individuals with low psychological adjustment, on the other hand, perceive challenging life events as more threatening and difficult to control. These evaluation patterns can make it difficult to develop a positive stress mindset, which includes beliefs about the management and potentially developmental aspects of stress. Furthermore, having negative cognitions in the face of stressful situations can lead to increased psychological pain (S. Chen et al., 2023). Indeed, a positive stress mindset contributes to a decrease in perceived stress levels by reducing the tendency to exaggerate challenges and perceive coping resources as insufficient. Increased perceived stress can strain an individual’s cognitive and emotional resources, reducing their capacity to regulate and tolerate psychological pain. Becker et al. (2019) also found that tolerance for psychological pain decreases as perceived stress increases. Consequently, it appears that psychological adjustment influences tolerance for psychological pain through cognitive evaluations and perceptions related to stress. Stress mindset and perceived stress function as fundamental psychological mechanisms in explaining this relationship.

Türkiye, the country where the research data was collected, recently experienced an earthquake disaster. Furthermore, the effects of economic instability and regional conflicts can lead to an intense and chronic social stress environment. The existing literature shows that such multiple stressors have profound effects on individuals’ psychological health. For example, a meta-analysis conducted after the earthquake found that post-traumatic stress symptoms remained high for a year (Çınaroğlu et al., 2025). In addition, economic uncertainties (Sarı et al., 2024) and proximity to conflict zones (Hoffmann et al., 2024) are known to be associated with the prevalence of social mental health problems. The findings obtained in the present research reflect the potential impact of this context on psychological processes. Individuals living in high-stress environments may become more vulnerable to stressful situations because their primary sources of psychological adjustment are damaged. This can lead to a chronic increase in perceived stress levels. Indeed, in our model, the strong role of psychological adjustment in reducing perceived stress is highlighted. Furthermore, the persistence of chronic social stress leads to individuals’ perceived stress levels remaining high for extended periods. The effect of perceived stress on reducing tolerance for psychological pain is clearly demonstrated in the findings of the present study. Therefore, this context may have provided an important framework for understanding the functioning of our model. In particular, it has made the impact of high-stress conditions on psychological mechanisms more pronounced. The research findings demonstrate, based on data, the importance of maintaining psychological adjustment and interventions targeting perceived stress in developing tolerance for psychological pain under challenging social conditions.

The findings of the study offer important clues about which variable interventions can be more effective. The direct effect of psychological adjustment level on perceived stress is stronger than the indirect effect caused by stress mindset. However, one of the pathways with the highest impact in the model is the pathway between perceived stress and tolerance for psychological pain. Considering all these findings together, preventive or interventional studies aimed at increasing tolerance to psychological pain should first help individuals acquire concrete skills to reduce their perceived stress levels. Furthermore, it is expected that developing interventions aimed at reducing perceived stress levels by transforming the stress mindset will contribute to the practical applicability of the research findings. Accordingly, instead of long-term interventions aimed at comprehensively altering psychological adjustment, it is recommended to develop more concrete and short-term interventions focused on regulating perceived stress. This is thought to create a faster and more direct impact on tolerance for psychological pain.

## 5. Limitations

This study has some limitations. First, since the data were collected with a cross-sectional design, causal relationships between variables cannot be determined with certainty. Therefore, longitudinal or experimental studies are needed in the future to more clearly demonstrate whether psychological adjustment increases psychological pain tolerance and in which direction this relationship operates. Second, the data were collected with self-report instruments. Participants’ self-reported responses may be biased due to social desirability effects or a lack of insight. This may cause the relationships between the variables to appear stronger or weaker than they are. Third, because the sample was limited to adults, the findings cannot be generalized to other age groups (under 18 years). Moreover, the study sample is limited to adults living in Turkey. As differences between countries may influence the psychological variables examined, the direct generalization of the findings to other cultural and national contexts is limited. The effects of age, socioeconomic status, and cultural differences on psychological adjustment, stress perception, and tolerance for psychological pain may differ. Therefore, studies conducted in different age groups and cultural contexts can test the validity of these findings.

In addition, all variables in this study were assessed using self-report measures and collected at a single time point. Therefore, the findings may be subject to common method variance, which could have inflated the observed associations among the variables. As no specific procedural or statistical remedies (e.g., marker variables or method bias tests) were employed to control for this potential bias, the results should be interpreted with this limitation in mind. In addition, use of the Brief Adjustment Scale-6 beyond its original student population should be considered when interpreting the results. Notably, the sample includes individuals who have only graduated from high school. Therefore, the way high school graduates and university and postgraduate students understand and interpret psychological adjustment may differ. This situation may lead to significant differences in the research results. In addition to differences in educational levels, the sample also shows differences in economic levels. Having different economic levels can be a determining factor in how stress is perceived. Especially considering the economic crisis and unemployment problem experienced in Türkiye in recent years, individuals’ employment status may also play an important role in coping with stress. Furthermore, the fact that the students are in the quarter-life crisis age range (18–30) may affect their chronic stress, psychological adjustment skills, and coping levels with psychological pain. Therefore, in future studies, the educational levels and socioeconomic status of the participants should be classified and addressed in more detail.

Finally, the study examined only stress mindset and perceived stress variables as mediators. However, other individual or environmental factors that may affect tolerance for psychological pain (e.g., age, gender, socioeconomical level, coping strategies, social support, traumatic past experiences) were not taken into account. Future studies may reach more comprehensive conclusions by incorporating these variables into the model. Furthermore, the conceptual relationships between psychological adjustment, perceived stress, and stress mindset variables can complicate research results. Particularly in Türkiye, where stress is generally perceived negatively, the concept of stress mindset, which interprets stress as developmental, may not have been fully understood by participants. Similarly, it can be assumed that perceived stress and debilitating stress mindset measure closely related constructs. Psychological adjustment, considering its ability to functionally adapt to stressful situations, can also be confused with stress mindset. Despite all these limitations, psychological adjustment, perceived stress, and stress mindset have different theoretical frameworks and explanations. In future studies on these concepts, whose distinctions were explained in the introduction, these limitations can be minimized by providing detailed explanations of the questionnaire items used to measure these concepts to the participants.

## 6. Conclusions

This study has revealed the determining effect of psychological adjustment on tolerance for psychological pain and how this relationship is shaped by stress mindset and perceived stress. The findings show that individuals with high psychological adjustment evaluate stressful situations more positively and functionally, thus decreasing their perceived stress levels and developing a higher resilience against psychological pain. The fact that stress mindset and perceived stress play a chain mediating role in the effect between psychological adjustment and tolerance for psychological pain emphasizes the importance of individuals’ cognitive and emotional stress-related processes. These results indicate that interventions to strengthen psychological adjustment can increase individuals’ psychological resilience against challenging life events by positively affecting stress perception and coping attitudes. In addition, it is understood that to reduce the risk of suicide and support psychological well-being, not only traumatic experiences but also the individual’s mindset and perceptions related to stress should be taken into consideration. It is recommended that these mechanisms be examined in different age groups and cultural contexts in future studies.

## 7. Implications

Before discussing the practical implications of the research findings, it is important to understand the practical function of the concept of tolerance for psychological pain. Tolerance for psychological pain does not mean passive helplessness in the face of pain. On the contrary, when an individual has a high tolerance for psychological pain, they can cope with it and mitigate its effects. The pain management aspect is one of the active coping strategies. The pain endurance aspect is fueled by the belief that the pain is temporary and bearable. Based on this conceptual clarity, there are some practical implications derived from the findings of the study. Psychoeducational or intervention programs designed to increase tolerance for psychological pain should have a holistic structure. The content of these programs should simultaneously consider interconnected goals such as psychological adjustment, stress mindset, and perceived stress. Intervention programs should aim to increase individuals’ level of psychological adjustment and transform their stress mindset. In this way, the individual’s capacity to cope with psychological pain can be improved by reducing their daily stress load (perceived stress). Stress mindset and perceived stress are also decisive in how individuals interpret and perceive stressful situations. Therefore, cognitive restructuring, emotion regulation, and mindfulness-based interventions can be effective in managing psychological distress functionally. When mental health professionals work with clients experiencing psychological pain, they should not focus solely on the clients’ levels of tolerance for psychological distress. Planning should consider variables such as adaptation, perceived stress, and stress mindset in order to adopt a holistic approach to managing psychological distress. Furthermore, school counselors can plan preventative interventions by assessing students’ tolerance for psychological pain, stress mindset, and perceived stress levels together. This can help students cope with stressful or challenging situations in a more adaptable and resilient way.

## Figures and Tables

**Table 1:** Demographic characteristics of participants (N = 709).

Variable	Category	n	%
Gender	Male	241	34.0
	Female	468	66.0
Age	Range	18–51	
	Mean (SD)	26.08 (6.41)	
Education Level	High school	133	18.8
	Bachelor’s degree	507	71.5
	Postgraduate degree	69	9.7
Socioeconomic Status	Poor	126	17.8
	Moderate	547	77.2
	Good	36	5.1

**Table 2:** Descriptive statistics and correlations.

	1	2	3	4
1 = Tolerance for psychological pain	1			
2 = Psychological adjustment	0.46 **	1		
3 = Stress mindset	0.14 *	0.10 **	1	
4 = Perceived stress	−0.51 **	−0.68 *	−0.14	1
Mean	32.22	24.91	8.66	21.00
Std. deviation	7.04	9.49	6.60	6.80
Skewness	−0.00	−0.15	0.45	0.05
Kurtosis	0.30	−0.67	−0.43	0.23

* *p* < 0.01; ** *p* < 0.05.

**Table 3:** Mediational model coefficients.

	Stress Mindset		Perceived Stress		Tolerance for Psychological Pain	
Predictors	*β*	*p*	*β*	*p*	*β*	*p*
Psychological adjustment	0.07	0.001	−0.48	0.001	0.16	0.001
Stress mindset	------	------	−0.07	0.005	0.07	0.005
Perceived stress	------	------	------	------	−0.36	0.005
Constant	6.92	0.001	33.55	0.001	35.09	0.005
	*R* = 0.10, *R*^2^ = 0.010		*R* = 0.68, *R*^2^ = 0.46		*R* = 0.54; *R*^2^ = 0.29	
	*F*(1, 707) = 7.215		*F*(2, 706) = 302.2		*F*(3, 705) = 95.84	
	Estimates of Point *β*		The lowest		The highest	
Ind1	0.005		0.001		0.012	
Ind2	0.17		0.12		0.22	
Ind3	0.0018		0.0010		0.004	

Ind1 = psychological adjustment → stress mindset → tolerance for psychological pain; Ind2 = psychological adjustment → perceived stress → tolerance for psychological pain; Ind3 = psychological adjustment → stress mindset → perceived stress → tolerance for psychological pain.

**Table 4:** Results of bootstrapping analyses for the chain mediation model.

Model Pathways	Coefficient	Confidence Interval (%95)	
		Lower	Upper
a → b	0.16	0.10 **	0.23 **
a → c	0.07	0.017 *	0.12 *
a → d	−0.48	−0.52 *	−0.44 *
c → d	−0.07	−0.13 *	−0.011 *
c → b	0.07	0.006 *	0.14 *
d → b	−0.36	−0.45 *	−0.26 *

a: psychological adjustment; b: tolerance for psychological pain; c: stress mindset; d: perceived stress. * *p* < 0.01; ** *p* < 0.05.

## Data Availability

The data presented in this study are available on request from the corresponding author due to privacy restrictions. The data were anonymized, ensuring that there was no breach of privacy. They will be shared in a manner that respects ethical protocols and data protection regulations. The dataset will be accessible only for academic purposes, and any use of the data will recognize the original study and maintain the confidentiality of the participants.

## References

[B1-behavsci-16-00151] Alker L. A. (2019). Distress tolerance as a mediator of the relation between stress mindset and anxiety. Bachelor’s thesis.

[B2-behavsci-16-00151] Arslan G., Türk N., Kaya A. (2024). Psychological vulnerability, emotional problems, and quality-of-life: Validation of the brief suicide cognitions scale for Turkish college students. Current Psychology.

[B3-behavsci-16-00151] Ballabrera Q., Gómez-Romero M. J., Chamarro A., Limonero J. T. (2024). The relationship between suicidal behavior and perceived stress: The role of cognitive emotional regulation and problematic alcohol use in Spanish adolescents. Journal of Health Psychology.

[B4-behavsci-16-00151] Bantjes J., Kagee A. (2018). Common mental disorders and psychological adjustment among individuals seeking HIV testing: A study protocol to explore implications for mental health care systems. International Journal of Mental Health Systems.

[B5-behavsci-16-00151] Bardeen J. R., Fergus T. A., Orcutt H. K. (2017). Examining the specific dimensions of distress tolerance that prospectively predict perceived stress. Cognitive Behaviour Therapy.

[B6-behavsci-16-00151] Başaran İ. E. (2005). Eğitim psikolojisi *[Educational psychology]*.

[B7-behavsci-16-00151] Batmaz H., Türk N., Doğrusever C. (2021). The mediating role of hope and loneliness in the relationship between meaningful life and psychological resilience in the COVİD-19 Pandemic during. Anemon Muş Alparslan Üniversitesi Sosyal Bilimler Dergisi.

[B8-behavsci-16-00151] Becker G., Orbach I., Mikulincer M., Iohan M., Gilboa-Schechtman E., Grossman-Giron A. (2019). Reexamining the mental pain–suicidality link in adolescence: The role of tolerance for mental pain. Suicide and Life-Threatening Behavior.

[B9-behavsci-16-00151] Bergin A. J., Pakenham K. I. (2016). The stress-buffering role of mindfulness in the relationship between perceived stress and psychological adjustment. Mindfulness.

[B10-behavsci-16-00151] Birman D., Simon C. D., Chan W. Y., Tran N. (2014). A life domains perspective on acculturation and psychological adjustment: A study of refugees from the former Soviet Union. American Journal of Community Psychology.

[B11-behavsci-16-00151] Bjørndal L. D., Ebrahimi O. V., Røysamb E., Karstoft K. I., Czajkowski N. O., Nes R. B. (2024). Stressful life events exhibit complex patterns of associations with depressive symptoms in two population-based samples using network analysis. Journal Of Affective Disorders.

[B12-behavsci-16-00151] Black J. S., Gregersen H. B. (1991). Antecedents to cross-cultural adjustment for expatriates in Pacific Rim assignments. Human Relations.

[B13-behavsci-16-00151] Bretherton S. J., McLean L. A. (2015). Interrelations of stress, optimism and control in older people’s psychological adjustment. Australasian Journal on Ageing.

[B14-behavsci-16-00151] Büyüköztürk Ş., Kılıç Çakmak E., Akgün Ö. E., Karadeniz Ş., Demirel F. (2014). Eğitimde bilimsel araştırma yöntemleri *[Scientific research methods in education]*.

[B15-behavsci-16-00151] Chen L., Qu L., Hong R. Y. (2022). Pathways linking the big five to psychological distress: Exploring the mediating roles of stress mindset and coping flexibility. Journal of Clinical Medicine.

[B16-behavsci-16-00151] Chen S., Cheng Y., Zhao W., Zhang Y. (2023). Psychological pain in depressive disorder: A concept analysis. Journal of Clinical Nursing.

[B17-behavsci-16-00151] Chen Y. L., Kuo P. H. (2020). Effects of perceived stress and resilience on suicidal behaviours in early adolescents. European Child & Adolescent Psychiatry.

[B18-behavsci-16-00151] Cohen S., Kamarck T., Mermelstein R. (1983). A global measure of perceived stress. Journal of Health and Social Behavior.

[B19-behavsci-16-00151] Conejero I., Olié E., Calati R., Ducasse D., Courtet P. (2018). Psychological pain, depression, and suicide: Recent evidences and future directions. Current Psychiatry Reports.

[B20-behavsci-16-00151] Crum A. J., Akinola M., Martin A., Fath S. (2017). The role of stress mindset in shaping cognitive, emotional, and physiological responses to challenging and threatening stress. Anxiety, Stress, & Coping.

[B21-behavsci-16-00151] Crum A. J., Salovey P., Achor S. (2013). Rethinking stress: The role of mindsets in determining the stress response. Journal of Personality and Social Psychology.

[B22-behavsci-16-00151] Cruz R. A., Peterson A. P., Fagan C., Black W., Cooper L. (2020). Evaluation of the brief adjustment scale–6 (BASE-6): A measure of general psychological adjustment for measurement-based care. Psychological Services.

[B23-behavsci-16-00151] Çetiner E., Sayın-Karakaş G., Selçuk O. C., Şakiroğlu M. (2018). Perceived stress and university adaptation process: The mediating role of mindfulness. Nesne Journal of Psychology.

[B24-behavsci-16-00151] Çınaroğlu M., Yılmazer E., Noyan Ahlatcioglu E., Ülker S. V., Hızlı Sayar G. (2025). Psychological impact of the 2023 Kahramanmaraş earthquakes: A systematic review and meta-analysis of PTSD, depression, and anxiety among Turkish adults. Frontiers in Public Health.

[B25-behavsci-16-00151] Demirkol M. E., Tamam L., Namlı Z., Eriş Davul Ö. (2019). Validity and reliability study of the Turkish version of the tolerance for mental pain scale-10. Psychiatry and Clinical Psychopharmacology.

[B26-behavsci-16-00151] Doğrusever C., Türk N., Batmaz H. (2022). The mediating role of meaningful life in the relationship between self-esteem and psychological resilience. İnönü Üniversitesi Eğitim Fakültesi Dergisi.

[B27-behavsci-16-00151] Ducasse D., Holden R. R., Boyer L., Artero S., Calati R., Guillaume S., Olie E. (2017). Psychological pain in suicidality: A meta-analysis. The Journal of Clinical Psychiatry.

[B28-behavsci-16-00151] Elbogen E. B., Lanier M., Blakey S. M., Wagner H. R., Tsai J. (2021). Suicidal ideation and thoughts of self-harm during the COVID-19 pandemic: The role of COVID-19-related stress, social isolation, and financial strain. Depression and Anxiety.

[B29-behavsci-16-00151] Eskin M., Harlak H., Demirkıran F., Dereboy Ç. (2013). Algılanan stres ölçeğinin Türkçeye uyarlanması: Güvenirlik ve geçerlik analizi. New Symposium Journal.

[B30-behavsci-16-00151] Garnefski N., Koopman H., Kraaij V., ten Cate R. (2009). Brief report: Cognitive emotion regulation strategies and psychological adjustment in adolescents with a chronic disease. Journal of Adolescence.

[B31-behavsci-16-00151] Hatun O., Kurtça T. T. (2024). Perceived Stress, hope, and life satisfaction among college students: A two-wave, half-longitudinal study from Turkey. International Journal of Mental Health and Addiction.

[B32-behavsci-16-00151] Hoffmann K., Michalak M., Kopciuch D., Bryl W., Kus K., Nowakowska E., Paczkowska A. (2024). The prevalence and correlates of anxiety, stress, mood disorders, and sleep disturbances in Poland after the outbreak of the Russian-Ukrainian War 2022. Healthcare.

[B33-behavsci-16-00151] Huebschmann N. A., Sheets E. S. (2020). The right mindset: Stress mindset moderates the association between perceived stress and depressive symptoms. Anxiety, Stress, & Coping.

[B34-behavsci-16-00151] Joffe W. G., Sandler J. (1967). On the concept of pain, with special reference to depression and psychogenic pain. Journal of Psychosomatic Research.

[B35-behavsci-16-00151] Journault A. A., Lupien S. J. (2024). Stress mindsets matter: An overview of how individuals think about stress, its effect on biopsychosocial processes, and what we can do about it. Psychoneuroendocrinology.

[B36-behavsci-16-00151] Keech J. J., Hamilton K., Gelman M. D. (2019). Stress mindset. Encyclopedia of behavioral medicine.

[B37-behavsci-16-00151] Klussman K., Lindeman M. I. H., Nichols A. L., Langer J. (2021). Fostering stress resilience among business students: The role of stress mindset and self-connection. Psychological Reports.

[B38-behavsci-16-00151] Laferton J. A., Fischer S., Ebert D. D., Stenzel N. M., Zimmermann J. (2020). The effects of stress beliefs on daily affective stress responses. Annals of Behavioral Medicine.

[B39-behavsci-16-00151] Lazarus R. S., Folkman S. (1984). Stress, appraisal, and coping.

[B40-behavsci-16-00151] Lecic-Tosevski D., Vukovic O., Stepanovic J. (2011). Stress and personality. Psychiatriki.

[B41-behavsci-16-00151] Levi-Belz Y., Gavish-Marom T., Barzilay S., Apter A., Carli V., Hoven C., Sarchiapone M., Wasserman D. (2019). Psychosocial factors correlated with undisclosed suicide attempts to significant others: Findings from the adolescence SEYLE study. Suicide and Life-Threatening Behavior.

[B42-behavsci-16-00151] Lindert J., Lee L. O., Weisskopf M. G., McKee M., Sehner S., Spiro A. (2020). Threats to belonging—Stressful life events and mental health symptoms in aging men—A longitudinal cohort study. Frontiers in Psychiatry.

[B43-behavsci-16-00151] Mansell P. C. (2021). Stress mindset in athletes: Investigating the relationships between beliefs, challenge and threat with psychological wellbeing. Psychology of Sport and Exercise.

[B44-behavsci-16-00151] Meerwijk E. L., Mikulincer M., Weiss S. J. (2019). Psychometric evaluation of the tolerance for mental pain scale in United States adults. Psychiatry Research.

[B45-behavsci-16-00151] Miron J., Goldberg X., López-Sola C., Nadal R., Armario A., Andero R., Giraldo J., Ortiz J., Cardoner N., Palao D. (2019). Perceived stress, anxiety and depression among undergraduate students: An online survey study. Journal of Depression and Anxiety.

[B46-behavsci-16-00151] Onan N., Barlas G. Ü. L., Karaca S., Yildirim N., Taskiran O., Sumeli F. (2015). The relations between perceived stress, communication skills and psychological symptoms in oncology nurses. Clinical and Experimental Health Sciences.

[B47-behavsci-16-00151] Orbach I., Mikulincer M., Sirota P., Gilboa-Schechtman E. (2003). Mental pain: A multidimensional operationalization and definition. Suicide and Life-Threatening Behavior.

[B48-behavsci-16-00151] Osaki Y., Otsuki H., Imamoto A., Kinjo A., Fujii M., Kuwabara Y., Kondo Y., Suyama Y. (2021). Suicide rates during social crises: Changes in the suicide rate in Japan after the Great East Japan earthquake and during the COVID-19 pandemic. Journal of Psychiatric Research.

[B49-behavsci-16-00151] Özgüven E. (1992). Hacettepe personality inventory handbook.

[B50-behavsci-16-00151] Park D., Yu A., Metz S. E., Tsukayama E., Crum A. J., Duckworth A. L. (2019). Beliefs about stress attenuate the relation among adverse life events, perceived distress, and self-control. Child Development.

[B51-behavsci-16-00151] Qiang S., Wu J., Zheng D., Xu T., Hou Y., Wen J., Liu J. (2025). The effect of stress mindset on psychological pain: The chain mediating roles of cognitive reappraisal and self-identity. Frontiers in Psychology.

[B52-behavsci-16-00151] Republic of Türkiye Ministry of Interior (2024). Türkiye’s strength in unity and solidarity was tested by the earthquake; The disaster of the century transformed into the solidarity of the century!.

[B53-behavsci-16-00151] Rohner R. P. (1975). They Love Me, They Love Me Not: A worldwide study of the effects of parental acceptance and recetion.

[B54-behavsci-16-00151] Rohner R. P. (1986). The warmth dimension: Foundations of parental acceptance-rejection theory.

[B55-behavsci-16-00151] Rohner R. P., Britner P. A. (2002). Worldwide mental health correlates of parental acceptance-rejection: Review of cross-cultural and intracultural evidence. Crosscultural Research.

[B56-behavsci-16-00151] Rohner R. P., Saavedra J. M., Granum E. O. (1978). Development and validation of the personality assessment questionnaire: Test manual.

[B57-behavsci-16-00151] Ruiz-Fernández M. D., Ramos-Pichardo J. D., Ibáñez-Masero O., Cabrera-Troya J., Carmona-Rega M. I., Ortega-Galán Á. M. (2020). Compassion fatigue, burnout, compassion satisfaction and perceived stress in healthcare professionals during the COVID-19 health crisis in Spain. Journal of Clinical Nursing.

[B58-behavsci-16-00151] Sarı E., Karakuş B. Ş., Demir E. (2024). Economic uncertainty and mental health: Global evidence, 1991 to 2019. SSM-Population Health.

[B59-behavsci-16-00151] Satıcı B., Gocet-Tekin E., Deniz M., Satici S. A., Yilmaz F. B. (2024). Mindfulness and well-being: A longitudinal serial mediation model of psychological adjustment and COVID-19 Fear. Journal of Rational-Emotive & Cognitive-Behavior Therapy.

[B60-behavsci-16-00151] Seaton C. L., Lopez S. J. (2009). Psychological adjustment. The encyclopedia of positive psychology.

[B61-behavsci-16-00151] Sehlikoğlu Ş., Yıldız S., Kurt O., Emir B. S., Sehlikoğlu K. (2024). Investigation of suicide attempt, impulsivity, psychological pain and depression in earthquake survivors affected by the February 6, 2023 Kahramanmaraş-centered earthquake. Journal of Harran University Medical Faculty.

[B62-behavsci-16-00151] Shelef L., Fruchter E., Hassidim A., Zalsman G. (2015). Emotional regulation of mental pain as moderator of suicidal ideation in military settings. European Psychiatry.

[B63-behavsci-16-00151] Silva F. C., Ferrreira M. T., Souza-Talarico J. N. (2023). Mapping stress-mindset definitions, measurements and associated factors: A scope review. Psychoneuroendocrinology.

[B64-behavsci-16-00151] Smith E. E., Hoeksema-Nolen S., Fredrickson B., Loftus G. R. (2016). Atkinson and Hilgard’s introduction to psychology.

[B65-behavsci-16-00151] Spătaru B., Podină I. R., Tulbure B. T., Maricuțoiu L. P. (2024). A longitudinal examination of appraisal, coping, stress, and mental health in students: A cross-lagged panel network analysis. Stress Health.

[B66-behavsci-16-00151] Subhasree G., Jeyavel S., Eapen J. C., Deepthi D. P. (2023). Stress mindset as a mediator between self-efficacy and coping styles. Cogent Psychology.

[B67-behavsci-16-00151] Sun X., Li B. J., Zhang H., Zhang G. (2023). Social media use for coping with stress and psychological adjustment: A transactional model of stress and coping perspective. Frontiers in Psychology.

[B68-behavsci-16-00151] Sutin A. R., Luchetti M., Stephan Y., Sesker A. A., Terracciano A. (2023). Purpose in life, stress mindset, and perceived stress: Test of a mediational model. Personality and Individual Differences.

[B69-behavsci-16-00151] Tamizi N. M. B. M., Perveen A., Hamzah H., Folashade A. T. (2024). Relationship of psychological adjustment, anxiety, stress, depression, suicide ideation, and social support among university students. International Journal of Academic Research in Business & Social Sciences.

[B70-behavsci-16-00151] Tehrani N. A., Bayazi M. H., Shakerinasab M. (2020). The relationship between islamic coping methods and psychological well-being with adaptation and pain tolerance in patients with breast cancer. Quarterly Journal of Health Psychology.

[B71-behavsci-16-00151] Turgut M. N. A., Ertürk Ö. S., Karslı F., Şakiroğlu M. (2020). A mediation variable in relation to perceived stres and adaptation to university life: Separation anxiety. Hacettepe University Journal of Education.

[B72-behavsci-16-00151] Turkish Statistical Institute (2024). İç göç istatistikleri [Internal migration statistics], 2023.

[B73-behavsci-16-00151] Türk N., Arslan G., Kaya A., Güç E., Turan M. E. (2024a). Psychological maltreatment, meaning-centered coping, psychological flexibility, and suicide cognitions: A moderated mediation model. Child Abuse & Neglect.

[B74-behavsci-16-00151] Türk N., Çelik M. (2024). Psychometric properties of the Turkish adaptation of stress mindset measure. Current Approaches in Psychiatry.

[B75-behavsci-16-00151] Türk N., Özmen M., Derin S. (2025a). Psychological strain and suicide rumination among university satudents: Exploring the mediating and moderating roles of depression, resilient coping, and perceived social support. Healthcare.

[B76-behavsci-16-00151] Türk N., Yasdiman M. B., Kaya A. (2024b). Defeat, entrapment and suicidal ideation in a Turkish community sample of young adults: An examination of the Integrated Motivational-Volitional (IMV) model of suicidal behaviour. International Review of Psychiatry.

[B77-behavsci-16-00151] Türk N., Yildirim M., Batmaz H., Aziz I. A., Gómez-Salgado J. (2025b). Resilience and meaning-centered coping as mediators in the relationship between life satisfaction and posttraumatic outcomes among earthquake survivors in Turkey. Medicine.

[B78-behavsci-16-00151] Weiten W., Yost Hammer E., Dunn D. S. (2011). Psychology and contemporary Life: Human adjustment.

[B79-behavsci-16-00151] Williams S. E., Ginty A. T. (2024). A stress-is-enhancing mindset is associated with lower traumatic stress symptoms during the COVID-19 pandemic. Anxiety, Stress, & Coping.

[B80-behavsci-16-00151] World Bank (2025). Fragile and conflict-affected situations: Intertwined crises, multiple vulnerabilities *[Press release]*.

[B81-behavsci-16-00151] World Economic Forum (2025). The global risks report 2025: 20th edition (Insight report).

[B82-behavsci-16-00151] World Health Organization (2025). Health: Who is health emergency appeal 2025.

[B83-behavsci-16-00151] Yeşiloğlu C., Tamam L., Demirkol M. E., Namlı Z., Karaytuğ M. O. (2023). Associations between the suicidal ideation and the tolerance for psychological pain and tolerance for physical pain in patients diagnosed with major depressive disorder. Neuropsychiatric Disease and Treatment.

[B84-behavsci-16-00151] Yıldırım M., Solmaz F. (2020). Testing a Turkish adaption of the brief psychological adjustment scale and assessing the relation to mental health. Studies in Psychology.

[B85-behavsci-16-00151] Yıldırım O., Batmaz H., Türk N. (2026). Parallel mediation of psychological flexibility and vulnerability between multiple-screen addiction and mental health outcomes in adolescents. BMC Psychology.

[B86-behavsci-16-00151] Zandifar A., Badrfam R., Yazdani S., Arzaghi S. M., Rahimi F., Ghasemi S., Khamisabadi S., Khonsari N. M., Qorbani M. (2020). Prevalence and severity of depression, anxiety, stress and perceived stress in hospitalized patients with COVID-19. Journal of Diabetes & Metabolic Disorders.

[B87-behavsci-16-00151] Zeng W., Ma S., Xu Y., Wang R. (2024). The roles of stress mindset and personality in the impact of life stress on emotional well-being in the context of COVID-19 confinement: A diary study. Appl Psychol Health Well Being.

[B88-behavsci-16-00151] Zhang N., Bai B., Zhu J. (2023). Stress mindset, proactive coping behavior, and posttraumatic growth among health care professionals during the COVID-19 pandemic. Psychological Trauma: Theory, Research, Practice, and Policy.

[B89-behavsci-16-00151] Zhao S., Li X., Li Y., Cao Y., Mi G., Chen L., Ye Z., Niu L. (2025). Reframing stress: The impact of stress mindset on adolescent sleep health. Journal of Research on Adolescent.

[B90-behavsci-16-00151] Zheng X., Sang D., Wang L. (2004). Acculturation and subjective well-being of Chinese students in Australia. Journal of Happiness Studies.

